# T Cell Epitope Peptide Therapy for Allergic Diseases

**DOI:** 10.1007/s11882-015-0587-0

**Published:** 2016-01-14

**Authors:** Robyn E. O’Hehir, Sara R. Prickett, Jennifer M. Rolland

**Affiliations:** Department of Allergy, Immunology and Respiratory Medicine, Alfred Hospital and Central Clinical School, Monash University, Commercial Road, Melbourne, Victoria 3004 Australia; Department of Immunology, Monash University, Melbourne, Victoria Australia

**Keywords:** T cell epitope, Peptide, SPIRE, Synthetic peptide immuno-regulatory epitopes, Allergy, Immunotherapy

## Abstract

Careful selection of dominant T cell epitope peptides of major allergens that display degeneracy for binding to a wide array of MHC class II molecules allows induction of clinical and immunological tolerance to allergen in a refined treatment strategy. From the original concept of peptide-induced T cell anergy arising from in vitro studies, proof-of-concept murine models and flourishing human trials followed. Current randomized, double-blind, placebo-controlled clinical trials of mixtures of T cell-reactive short allergen peptides or long contiguous overlapping peptides are encouraging with intradermal administration into non-inflamed skin a preferred delivery. Definitive immunological mechanisms are yet to be resolved but specific anergy, Th2 cell deletion, immune deviation, and Treg induction seem implicated. Significant efficacy, particularly with short treatment courses, in a range of aeroallergen therapies (cat, house dust mite, grass pollen) with inconsequential non-systemic adverse events likely heralds a new class of therapeutic for allergy, Synthetic Peptide Immuno-Regulatory Epitopes (SPIRE).

## Introduction

In 1911, Noon pioneered allergen immunotherapy (AIT) for treatment of grass pollen allergy [1]. The administration of allergen extracts using various regimens and routes is now established therapeutic practice in the management of allergic diseases, and currently the only therapy proven to modify the natural course of an allergic disease. However, treatment with whole allergen extracts is contraindicated in patients with unstable asthma and food allergies due to the risk of severe IgE-mediated adverse events including anaphylaxis or even death [2–4]. These risks, together with the typically prolonged and frequent dosing regimens, command poor adherence in real life situations [5] and less than desirable availability of specific allergy treatments for the burgeoning allergy epidemic [6].

The pivotal role of the CD4^+^ T cell in driving immune responsiveness to allergen, together with distinct differences between T cell and B cell epitopes, facilitates development of refined therapeutics for redirecting the cellular immune response towards peripheral tolerance [7, 8••]. Allergen T cell epitopes are typically short, without conformational structure. They fail to cross-link cell-bound IgE or activate inflammatory mast cells and basophils. In contrast, allergen B cell epitopes are usually conformational although linear epitopes are described. On native allergen molecules, B cell epitopes can bind and cross-link specific IgE on effector cells to trigger degranulation with inflammatory mediator release and synthesis eliciting the well-recognized features of allergy. Carefully selected dominant T cell epitope peptides of major clinically relevant allergens that display degeneracy for binding to a wide array of MHC class II molecules can safely induce clinical and immunological tolerance in a breakthrough new class of allergy treatment, recently termed Synthetic Peptide Immuno-Regulatory Epitopes (SPIRE) [8••, 9]. Evolution from the idea of allergen T cell epitope-based peptide anergy [10], through proof-of-concept murine experimental models [11–13], progressed to human clinical translation [14, 15]. In recent years, there have been randomized, double-blind, placebo-controlled clinical trials of two types of T cell epitope-based therapeutics for allergy: short T cell epitope peptide mixtures and longer Contiguous Overlapping Peptides (COPs) [9, 16•]. Significant efficacy has been achieved in cat, house dust mite (HDM), and pollen allergy with short treatment courses and inconsequential non-systemic adverse events [17, 18••, 19]. Underlying immunological mechanisms are yet to be clarified but likely include similar changes in allergen-specific T cell responses to those seen in conventional AIT [8••, 20••]. Whether induction of blocking IgG_4_ antibodies is required for efficacy of peptide immunotherapy is not so clear and may only be seen using longer peptides such as COPs or on re-exposure to native allergen [16•]. Promisingly, T cell epitope peptide therapies suggest efficacy with enhanced safety, allowing wider uptake across a range of allergic conditions.

The focus of this review is the rationale, design and utilization of T cell epitope-based peptides for specific treatment of allergic diseases. Selected peptides comprise immunodominant T cell epitopes but not IgE-binding epitopes and have minimal stimulatory potential for inflammatory cells. Their presentation is in a form that induces long-lasting allergen-specific T cell non-responsiveness after only a short course of treatment. Recent highly encouraging clinical trials of this new class of allergy therapy and associated data on immunological mechanisms are discussed.

## The Rationale for T Cell Epitope Peptide Therapy for Allergic Diseases

T cell epitope peptide therapy harnesses the established immunological dogma that *dominant T cell epitope peptides can induce anergy of specific T cells* if delivered in a way that fails to activate the T cell [21]. Induction of specific anergy utilizes the functional cytokine plasticity of Th cells to downregulate aberrant effector T cell responses while providing the cytokine milieu and impetus for naive T cells to establish protective responses [22, 23]. Additionally, the pattern of conserved T cell epitope repertoires observed in HDM-allergic individuals during longitudinal screening over 2 years supports this approach in contrast to the more changeable T cell specificities observed in longitudinal screening in autoimmune disease [24, 25]. Human T cell receptor repertoire analysis identified TCR-Vα and TCR-Vβ gene segment usage bias, together with in vivo *clonal dominance* by long-lived HDM-specific T cell clones [26]. Similar in vivo longevity of venom-specific T cell clones was reported [27]. Most importantly, *functional plasticity* of T cells derived from the same clonal origin allows *switching* from dominant IL-4 to IL-10 or IFN-γ production during anergy induction in vitro or AIT [27–29]. Another study demonstrated preferential loss (deletion) of pathogenic Th2 T cells specific for dominant allergen epitopes following successful pollen immunotherapy using fluorochrome-conjugated HLA class II-peptide tetramers to quantify these T cells directly ex vivo [30••]. These features support the view that induction of specific anergy or selective elimination of dominant clonal populations of pathogenic allergen-specific T cells would be of therapeutic benefit in the treatment of allergic diseases.

## Design of T Cell Epitope Peptide Therapies for Allergic Diseases

### T Cell Epitope Mapping of Major Allergens

T cell epitope peptide therapies rely on the identification of immunodominant CD4^+^ T cell epitopes within major, clinically relevant allergens. Molecular cloning, characterization and sequencing of allergens allow the synthesis of nested sets of overlapping peptides covering the full allergen sequence. Mapping of T cell epitopes can then be performed using peripheral blood mononuclear cells (PBMC) from individuals with the specific allergy of interest, either directly ex vivo, or after enrichment for allergen-specificity as T cell lines (oligoclonal populations) or T cell clones (monoclonal populations). The most critical peptides are identified by a range of immunological assays utilising sophisticated and/or high-throughput methodologies. These include flow cytometry using dyes such as carboxyfluorescein diacetate succinimidyl ester (CFSE) to detect proliferating cells by decreased intensity of staining [31, 32], cytokine capture [33], and fluorochrome-conjugated HLA class II-peptide tetramers [27, 34]. CFSE-based approaches are sensitive for detection of peptide-responsive T cells, particularly when combined with other activation markers such as CD25 (our unpublished observation), but bystander proliferation may reduce specificity [35•]. ELISPOT-based approaches can be used for high-throughput screening of PBMC for T cell epitope peptide recognition [33, 36, 37]. Identified T cell epitopes are then validated by screening large patient population cohorts and using rigorous assay design and appropriate statistical methods (e.g., [34]).

HLA-peptide tetrameric complexes are sensitive and specific analytes for identification and characterization of allergen-specific T cells directly ex vivo, but tetramer synthesis is expensive and many HLA class II molecules are not easily isolated for use in tetramers, limiting the HLA-coverage obtainable [27, 34]. Unlike CFSE-approaches, tetramer-based methodologies may lack sensitivity despite high specificity [35•]. Alternatively, in silico algorithms consider thousands of known epitope sequences to predict CD4^+^ T cell epitopes by detecting theoretical HLA class II binding motifs within protein sequences [38]. While algorithms provide preliminary guidance cost-effectively, comprehensiveness is limited and HLA-binding motif predictions require validation in functional peripheral blood T cell assays [35•, 39].

Most frequently, for identification of all potential T cell epitopes, allergen-specific T cell lines and clones from large patient cohorts are screened for reactivity against overlapping synthetic peptides spanning the entire sequence of the allergen molecule, each usually 15 to 20 amino acids long with overlaps from 5 amino acids upwards. Core epitopes within T cell-reactive peptides are mapped subsequently using peptide sets truncated from the N- and C-termini, typically revealing eight or nine residue core epitopes for CD4^+^ T cells. Optimal T cell stimulation often requires longer sequences including flanking residues to stabilize the HLA-peptide-TCR complex and improve expression of peptide on the antigen presenting cell surface [40–42]. Consistent with naturally processed peptides eluted from HLA class II molecules, candidate peptides for inclusion in short allergen peptide therapy range from 12–20 residues [43, 44].

A catalogue of allergen T cell-reactive sites mapped to date is available from The Immune Epitope Database (IEDB) [45–47]. Meta-analysis identified 1406 allergen-derived CD4^+^ T cell epitopes derived from human T cell reactivity [48]. Despite large numbers, it is estimated that this represents <17 % of all allergens in the International Union of Immunological Societies (IUIS) allergen database [49]. T cell epitopes are found scattered throughout an allergen sequence, but consideration of collective properties of the epitopes allows ranking according to dominance to optimize peptide candidates for therapy [38]. Such properties include donor and T cell line/clone responder frequency, patterns of reactivity, reproducibility of T cell response and, importantly, ability to induce a response in patient PBMC in large patient cohorts. Identification of the most immunodominant T cell epitopes is important, recognizing another important immunological dogma that *the stronger the immunogen the stronger the tolerogen* [50]. With neighboring or overlapping epitopes, a single peptide containing the two epitopes may allow targeting of both T cell specificities while minimising final peptide length and number. However, validation of T cell recognition of both epitopes in the consolidated peptide is required, and in our experience, not always obtained, requiring retention of individual T cell epitope peptides. Cross-reactive T cell epitopes are present in some closely related allergens, e.g. group 1 grass pollen allergens [51–53], which aids broader population coverage in different regions.

For optimal production, modification of some peptides may aid solubility and stability, for example, modification of terminal residues and replacement of cysteine residues with inert alanine or non-reactive serine to prevent aggregation (e.g. [32]). Testing T cell reactivity of any modified peptide is necessary to confirm retention of T cell stimulatory capacity. As a safety index, all candidate T cell epitope peptides need testing singly and in combination to exclude potential engagement and cross-linking of inflammatory cell-bound IgE. The flow cytometric basophil activation test or the histamine release test are increasingly utilized as convenient and validated read-outs of clinically relevant, functional IgE reactivity [54–57].

### MHC Class II Restriction Specificity

The ability of T cell epitope peptide candidates for therapy to show widespread degeneracy of binding to a range of MHC class II molecules is important when targeting genetically diverse patient populations. Human CD4^+^ T cells recognize allergen epitopes in the context of particular HLA class II molecules encoded by one of three highly polymorphic loci, HLA-DR, HLA-DP, or HLA-DQ. HLA-binding T cell epitope prediction algorithms are popular but with variable correlation when compared with direct functional assays. Early algorithms were limited to HLA-DR binding motifs, but now extend to HLA-DQ and -DP predictions [38]. Experimental validation of predicted epitopes utilizes isolated HLA molecules and/or transfected L cells or EBV-transformed B cell lines homozygous for defined HLA alleles [8••, 58]. HLA-peptide binding assays help inform clinical relevance in addition to levels of avidity and/or affinity [40, 58, 59].

However, to identify the full repertoire of functional HLA-peptide complexes, functional read-outs of T cell proliferation or cytokine production with the particular HLA-peptide complex are needed. Preliminary screening of HLA-restriction specificity of T cell epitope recognition is achieved by using blocking monoclonal antibodies specific for HLA-DR, HLA-DP or HLA-DQ [8••, 32]. HLA-genotyping of the antigen presenting cells can then inform restriction to specific HLA-subtype(s). Tetramers provide a more sophisticated method for screening in samples such as blood analyzed directly ex vivo [34] but, problematically, screens utilizing tetramers or homozygous cell lines need HLA-matched CD4^+^ T cells/patients [60] and as discussed above, the available range of testable HLA tetramers is limited.

It is helpful that allergen T cell epitopes frequently display extensive HLA-binding degeneracy while allergen-specific CD4^+^ T cells may recognize a particular epitope in the context of multiple HLA class II molecules [30••, 32, 33, 37, 38, 61–63]. Nominal antigens are most commonly presented on HLA-DR molecules, but allergen T cell epitopes are also presented on HLA-DQ and HLA-DP molecules (reviewed in [8••]). HLA-DQ and HLA-DP subtypes are often conserved more broadly across populations than HLA-DR molecules, for example HLA-DP*0401 and/or 0402 alleles are expressed in ~50 % of the Caucasian population [64].

## Learning from Experimental Models of Allergen T Cell Epitope Peptide Immune Modulation

In the early 1990s, O’Hehir and colleagues demonstrated that overnight incubation of cloned human T cells reactive with Der p 1 (major HDM allergen) with a supraoptimal concentration of their dominant T cell epitope peptide could induce specific non-responsiveness to that peptide [65]. The induction phase of anergy in this allergy model was associated with transient release of Th2 cytokines (notably the bronchoconstrictor IL-4) as though by hyper-excitation followed by anergy [8••, 28, 29]. Established anergy was accompanied by decreased IL-4 and IL-5 synthesis but maintained IFN-γ and IL-10 production [28, 65]. Defective TCR signalling was demonstrated by abrogated activity of p56^lck^ and ZAP-70 tyrosine kinases in a bee venom allergen (PLA2)-specific CD4^+^ T cell model [66].

Proof-of-concept for clinical translation was established initially using murine models of HDM and cat allergy [11, 12]. Importantly, these studies showed that delivering supraoptimal concentrations of a single dominant T cell epitope peptide in vivo not only induced specific non-responsiveness to subsequent challenge with that peptide, but also to whole allergen extract, known as linked suppression. Such linked epitope suppression confirmed in vivo applicability of allergen T cell epitope peptides to treat allergic disease. Subsequently, the robustness of this approach was validated in other allergen models (see review [8••]). Using HLA-DR1 tetramer technology in a murine cat allergy model, Campbell et al. identified Fel d 1 T cell epitope peptide-induced linked epitope suppression associated with IL-10^+^ T cells [67]. In a murine ovalbumin TCR transgenic model, Mackenzie et al. adoptively transferred Th2-polarized cells to show preferential effects of peptide immunotherapy on cytokine secretion by CD62L^lo^ cells (effector and effector memory T cells) rather than CD62L^hi^Th2 cells (central memory T cells) in downregulation of airway inflammation [68].

## Clinical Translation of T Cell Epitope Peptide Therapy for Allergy

### Learning from Early Trials of Allergen Peptides in Bee Venom and Cat Allergy

Pioneering in vivo studies in bee venom allergy confirmed that immunodominant T cell epitope peptides administered by subcutaneous injection induced clinical tolerance without severe adverse reactions [15]. However, these studies did not progress due to the unpredictable natural history of reactions to bee venom.

Pilot studies in cat allergy showed variable efficacy, but large protein determinants from Fel d 1 were trialled in these early studies rather than minimal epitopes [14, 69]. A double-blind placebo-controlled trial in 95 cat-allergic subjects comprising 4 subcutaneous doses of a Fel d 1 peptide mix (Allervax®CAT) or placebo showed clinical benefit of the peptide treatment after 6 weeks [14]. However, a range of adverse events was noted (nasal congestion, flushing, pruritus, chest tightness from minutes to hours after peptide delivery). Late asthmatic responses were triggered in some trial subjects with or without known asthma, and these were shown to be due to cytokines from peptide-stimulated T cells [70, 71]. It is tempting to speculate that the observed bronchospasm was due to the initial T cell activation and cytokine flare (particularly bronchoconstrictor IL-4) reported early in the induction phase of allergen peptide-induced anergy in vitro [28, 29]. Repeated delivery and dosage adjustment attenuated the late reaction without losing efficacy. Nevertheless, these early studies with higher concentrations of longer peptides administered subcutaneously were disappointing in failing to achieve sustained clinical efficacy [71, 72].

### Synthetic Peptide Immuno-Regulatory Epitope Therapy: a New Class of Anti-allergy Therapeutic

#### Short Allergen T Cell Epitope Peptides

Innovative research lead by Larché and Kay in the late 1990s and early 2000s evolved into second generation T cell epitope-based allergen peptides [70, 73]. Typically, these allergy therapeutic candidates are now mixtures of short peptides administered intradermally (≤12 nmol; ~75 μg vs 750 μg) into non-inflamed skin [9, 18••, 56, 73–76]. While earlier studies utilized the subcutaneous route for peptide administration, a subsequent study directly comparing intradermal with subcutaneous cat peptide delivery, showed that the intradermal route had superior immunological activity as well as tolerability [56]. The first of the new-generation peptide treatment to be trialled, Cat-PAD (Circassia Ltd; Oxford, UK), comprised of 7 T cell epitope-based peptides (13–17 amino acids) from Fel d 1as a lyophilisate reconstituted in water for intradermal administration. Recent trials have used a painless, needle-free transdermal delivery for four to eight treatments at 2–4-week intervals with recording of Total Rhino-conjunctivitis Symptom Score and challenge testing in an Environmental Exposure Chamber [18••, 77]. By using short T cell epitope-based peptides, IgE cross-linking and inflammatory cell activation was avoided, and careful dose adjustments prevented the late asthmatic responses seen earlier with longer peptides. A phase IIb clinical field trial indicated sustained clinical efficacy out to 2 years after the initial course of Cat-PAD [78••]. As expected in a clinical trial of an allergy therapy [79, 80], substantial placebo effects were seen initially but were not sustained longer term. Trials of short peptide therapies exploring optimal regimens in HDM [17, 81], grass pollen [82], and ragweed pollen allergy [83] are ongoing with encouraging early findings.

#### Contiguous Overlapping Peptides

In parallel, a second approach utilising longer T cell epitope-based allergen peptides, termed contiguous overlapping peptides (COPs), is under active investigation and early phase clinical trials [16•, 84, 85]. In this strategy, all possible T cell epitopes of the target allergen (birch pollen being the prototype) are included in a small set of long synthetic peptides but conformational IgE epitopes are disrupted. Phase 1/IIa clinical trials in bee venom and birch pollen allergy failed to invoke early IgE-mediated reactions and demonstrated increased specific IgG_4_ antibodies as observed with conventional AIT. A regimen tested in subjects with birch pollen allergy, comprising five injections in 2 months, resulted in improved nasal provocation scores. As seen in the early cat peptide trials, higher concentrations of COPs also invoked late asthmatic responses that were absent with lower peptide concentrations [16•]. It is again tempting to suggest that a surge of IL-4 release during the induction phase of anergy could explain the bronchoconstriction as a transient effect. While encouraging, further trial results for COPs are needed for convincing proof of efficacy.

## Analysis of Mechanisms of Action

As clinical translation of T cell epitope peptide therapy for allergy progresses, the underlying immunological mechanisms must be fully elucidated [20••, 86]. With increasing data, distinct differences are evident from conventional AIT [87, 88]. As seen in effective AIT with whole allergen, decreased T cell proliferative and cytokine response to allergen appear to follow allergen peptide immunotherapy (e.g., [72, 76]), but the precise mechanism is elusive to date. Some hallmarks of anergy are evident from the pilot bee venom study, in which decreased PLA2-specific T cell proliferation and decreased IL-2, IL-4, IL-5, IL-13, and IFN-γ production could be reversed by IL-2 and IL-15 [15]. However, in these early experiments, the polyclonal cultures and limited phenotyping precluded delineation of re-activated anergized T cells and activated naive T cells or even Treg. A role for deletion of allergen-specific T cells is also feasible from the murine models. In a recent study of conventional subcutaneous AIT for grass pollen allergy, HLA class II tetramers were used to quantify allergen-specific clonal T cell populations ex vivo [30••]. Findings were consistent with preferential loss of clonal Th2-type T cells specific for dominant epitopes of major grass pollen allergens over T cells specific for less-dominant epitopes with a Th-1 or Tr1-phenotype. However, tetrameric approaches rely on TCR detection on the cell surface which may be confounded by down-regulation of TCR expression on anergic T cells. In this study, the pathogenic Th2 cells also lacked CD27 expression as an additional marker suggesting selective loss of these cells.

Increased synthesis of IL-10 and induction of Treg are consistently reported as mechanisms of effective conventional AIT. Comparably, similar findings were reported in the early bee venom and cat allergen peptide studies [72, 89]. Specifically, the studies of cat allergen peptide treatment demonstrated a requirement for IL-10 in order to induce suppression of allergen-specific responses and invoke linked epitope suppression [67], and also to see an allergen-specific CD4^+^ Treg population [90]. Skin allergen challenge biopsies revealed enhanced CD4^+^IFN-γ^+^ and CD25^+^ cells following peptide treatment consistent with immune deviation and Tregs [91]. However, the overlap between surface markers on effector cells and Tregs, particularly when activated, makes interpretation of clinical biopsies difficult [92].

Peptide lengths selected for T cell epitope-based therapy may determine whether specific IgG_4_ blocking antibody is generated. Peptides based on individual minimal T cell epitope sequences are short and screened for lack of IgE binding and inflammatory cell activating potential, so would seem less likely to be immunogenic. Nevertheless, boosting by exposure to whole allergen in a modified cytokine milieu might conceivably drive IgA or IgG_4_ antibody production. The initial pilot bee venom study detected no antibody changes but, following subcutaneous rechallenge with whole allergen, an increase in specific IgG_4_ could be detected [15]. In a study by Tarzi et al., only a negligible and transient elevation in specific IgG_4_ was seen [89]. In contrast, repeated administration of COPs for birch pollen allergy resulted in a marked increase in Bet v 1-specific IgG_4_ levels over baseline and compared with placebo [16•]. As the data from current and future clinical trials accumulate, there will be greater appreciation of IgG_4_ changes as mechanistic or an epiphenomenon. Blocking antibodies are demonstrably important in conventional AIT but seem less relevant for sustained unresponsiveness [20••, 93].

## Conclusions

A new class of specific T cell epitope peptide therapy for allergic diseases, perhaps to be known as SPIRE, is growing in momentum with encouraging data in clinical trials across a range of allergens. Immunodominant T cell epitope-based allergen peptides seem able to induce sustained clinical tolerance although the underlying immunological mechanisms are yet to be clarified. Core epitope mapping to inform peptide selection identifies the critical amino acid sequences that can invoke desired clinical tolerance without undesirable cell-bound IgE cross-linking and inflammatory sequelae. Promiscuity of binding of these dominant allergen peptides to a wide range of HLA class II molecules offers widespread clinical utility without need for individual patient endotyping. In particular, emerging evidence of sustained efficacy in the presence of ongoing exposure to ensure retained memory after relatively short treatment courses without adverse events gives hope that allergology is poised for a therapeutic revolution. The observed pattern of efficacy suggests an important role for anergy as a key mechanism for this class of therapy. Expansion of this treatment approach to encompass specific therapies for food allergies, particularly peanut allergy, is advanced and early phase trials are awaited with interest. Additionally, research into possible applicability for mould allergy and cockroach allergy seems worthy of concentrated effort. After more than 100 years since specific immunotherapy was introduced as a treatment to modify the natural course of allergic disease, it is gratifying that harnessing fundamental immunological principles may allow more widespread modulation of the aberrant immune response to allergens that causes such a major socio-economic health burden globally.
